# Metabolic Analysis of Four Cultivars of *Liriope platyphylla*

**DOI:** 10.3390/metabo9030059

**Published:** 2019-03-26

**Authors:** Chang Ha Park, Abubaker Mohammed Awad Morgan, Byung Bae Park, Sook Young Lee, Sanghyun Lee, Jae Kwang Kim, Sang Un Park

**Affiliations:** 1 Department of Crop Science, Chungnam National University, 99 Daehak-ro, Yuseong-gu, Daejeon 34134, Korea; parkch804@gmail.com (C.H.P.); abubaker_morgan@yahoo.com (A.M.A.M.); 2 Department of Environment and Forest Resources, Chungnam National University, 99 Daehak-ro, Yuseong-gu, Daejeon 34134, Korea; bbpark@cnu.ac.kr; 3 Marine Bio Research Center, Chosun University, 61-220 Myeongsasimni, Sinji-myeon, Wando-gun, Jeollanamdo 59146, Korea; seedbank@chosun.ac.kr; 4 Department of Plant Science and Technology, Chung-Ang University, Anseong 456-756, Korea; slee@cau.ac.kr; 5 Division of Life Sciences and Bio-Resource and Environmental Center, Incheon National University, Incheon 406-772, Korea

**Keywords:** *Liriope platyphylla*, Liliaceae, spicatoside A, steroidal saponins, phenylpropanoid

## Abstract

*Liriope platyphylla* (Liliaceae), a medical plant distributed mainly in China, Taiwan, and Korea, has been used traditionally for the treatment of cough, sputum, asthma, and neurodegenerative diseases. The present study involved the metabolic profiling of this plant and reports spicatoside A accumulation in four different varieties of *L*. *platyphylla* (Cheongyangjaerae, Seongsoo, Cheongsim, and Liriope Tuber No. 1) using HPLC and gas chromatography time-of-flight mass spectrometry (GC–TOFMS). A total of 47 metabolites were detected in the different cultivars using GC–TOFMS-based metabolic profiling. The resulting data were subjected to principal component analysis (PCA) for determining the whole experimental variation, and the different cultivars were separated by score plots. Furthermore, hierarchical clustering, Pearson’s correlation, and partial least-squares discriminant analyses (PLS-DA) were subsequently performed to determine significant differences in the various metabolites of the cultivars. The HPLC data revealed that the presence of spicatoside A was detected in all four cultivars, with the amount of spicatoside A varying among them. Among the cultivars, Liriope Tuber No. 1 contained the highest amount of spicatoside A (1.83 ± 0.13 mg/g dry weight), followed by Cheongyangjaerae (1.25 ± 0.01 mg/g dry weight), Cheongsim (1.09 ± 0.04 mg/g dry weight), and Seongsoo (1.01 ± 0.02 mg/g dry weight). The identification of spicatoside A was confirmed by comparing the retention time of the sample with the retention time of the standard. Moreover, the Cheongsim cultivar contained higher levels of phenolic compounds—including vanillic acid, quinic acid, gallic acid, chlorogenic acid, caffeic acid, and benzoic acid—than those of the other two cultivars. On the other hand, the levels of amino acids were higher in the Seongsoo cultivar. Therefore, this study may help breeders produce new varieties with improved nutraceutical and nutritional qualities.

## 1. Introduction

*Liriope platyphylla* Wang et Tang, belonging to the Liliaceae family, is a herbaceous perennial plant, the members of which typically contain unusual and/or unique phytochemicals [1]. This medical plant is distributed mainly in China, Taiwan, and Korea, and has been used traditionally for the treatment of cough, sputum, asthma, and neurodegenerative diseases [2]. It has also been used traditionally as an antitussive agent, expectorant, and tonic in Korea [3]. Furthermore, a previous study reported that the compounds isolated from *L. platyphylla* possess anticancer properties, even if the mechanisms remain largely unknown [4].

Plant primary metabolism includes biological processes involved in the biosynthesis and use of a variety of endogenous building blocks such as nucleotides, amino acids, lipids, and carbohydrates, and energy sources contributing to the plant survival. On the other hand, plant secondary metabolism includes chemical reactions, which play major roles in plant development, defense, and reproduction, but are not essential for survival [5,6]. 

A phytochemical study revealed that *L. platyphylla* contains various steroidal saponins as major secondary metabolites. Of the known steroidal saponins, spicatoside A (25(*S*)-ruscogenin-1-*O*-*β*-D-glucopyranosyl(1→2)-[*β*-D-xylopyranosyl(1→3)]-*β*-D-fucopyranoside) was found to stimulate the secretion of growth hormone and induce neurite outgrowth [1]. Spicatoside A has been reported to have antiosteoarthritic activity, memory enhancement properties, and relieving effects in inflammatory pulmonary diseases [7]. However, the antitumor potential and its underlying mechanisms of action against human cancer cells have not been elucidated yet.

Phenolics, mainly biosynthesized from the phenylpropanoid biosynthesis pathway, are distributed in most plants and involved in protecting plants against biotic or abiotic stresses [8,9]. According to previous studies, phenolic compounds possess diverse biological activities, such as antioxidant [10], anti-inflammatory [11] and anti-cancer effects [12]. Therefore, intake of dietary phenolics is highly recommended for human health. 

Metabolic profiling has been used widely for providing biological information, through identification and quantitation of primary and secondary metabolites in plant systems [13,14,15]. Chromatography/mass spectrometry systems are suitable for metabolic profiling to detect and identify numerous metabolites by partial or full separation of the metabolites and their sensitive detection [16]. Among such systems, gas chromatography time-of-flight mass spectrometry (GC–TOFMS) has been employed to detect relatively low-molecular-weight metabolites (≤1000 Da) with fast scan times and high mass accuracy and resolution [17,18]. 

To our knowledge, no previous study has provided a comprehensive description of the primary metabolites, including carbohydrates, amino acids, and organic acids, and secondary metabolites, including spicatoside A and phenolics, in four different varieties of *L*. *platyphylla* (Cheongyangjaerae, Seongsoo, Cheongsim, and Liriope Tuber No. 1). Therefore, this study aims to gain a comprehensive understanding of the metabolic differences between the varieties of *L*. *platyphylla*. 

## 2. Results

### 2.1. Analysis of Spicatoside A

Using metabolic profiling, the present study investigated spicatoside A accumulation in four different varieties of *L*. *platyphylla* (Cheongyangjaerae, Cheongsim, Liriope Tuber No. 1, and Seongsoo). The HPLC data revealed the presence of spicatoside A in all four cultivars, with the amount of spicatoside A varying among them. Among the cultivars, Liriope Tuber No. 1 contained the highest amount of spicatoside A (1.83 ± 0.13 mg/g dry weight), followed by Cheongyangjaerae, Cheongsim, and Seongsoo (1.25 ± 0.01, 1.09 ± 0.04, and 1.01 ± 0.02 mg/g dry weight, respectively) (Table 1). The identification of spicatoside A was confirmed by comparing the retention time of the sample with the retention time of the standard. 

### 2.2. Analysis of Phenolic Compounds

A total of five phenolic compounds, including catechin, gallic acid, chlorogenic acid, caffeic acid, and benzoic acid, were identified and quantified in four different varieties of *L*. *platyphylla* by HPLC (Table 2). Cheongsim contained higher concentrations of total phenolics (461.11 ± 4.96 µg/g dry weight), which were 1.36 times higher than the concentrations found in Cheongyangjaerae (340.03 ± 4.29 µg/g dry weight). Moreover, a comparison of individual phenolics showed that Cheongsim contained higher amounts of gallic acid, caffeic acid, and chlorogenic acid than those of the other varieties, respectively. On the other hand, Liriope Tuber No. 1 contained a higher level of benzoic acid than the other varieties.

### 2.3. GC‒TOFMS‒Based Metabolic Profiling and Multivariate Analysis

**Figure 1:**
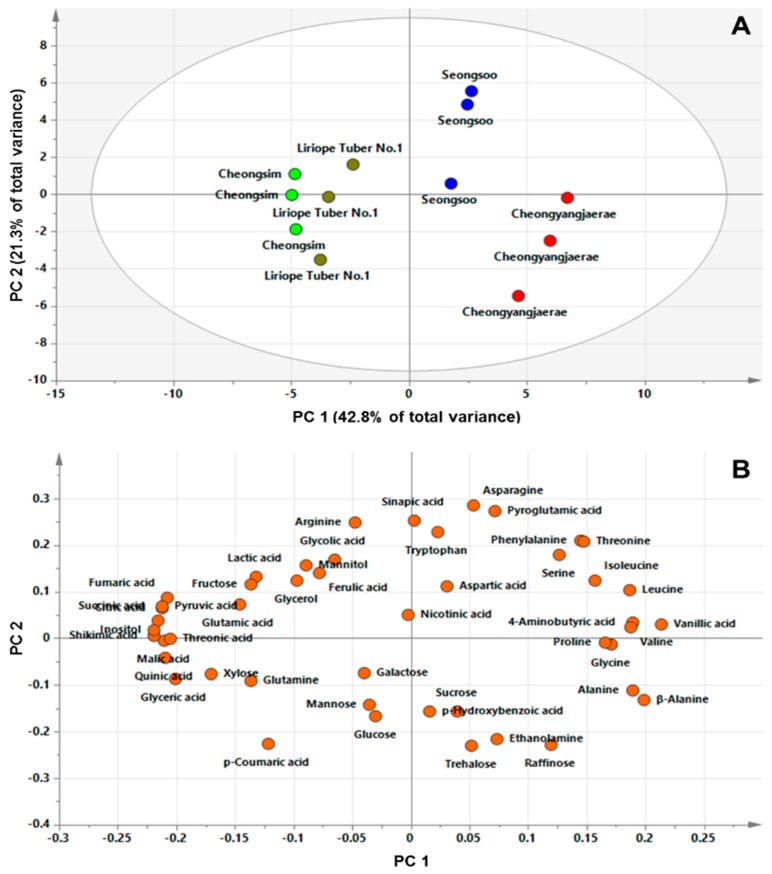
Principal component analysis (PCA) results obtained from data on 47 metabolites for four cultivars of *L. platyphylla* Wang et Tang. (**A**) Score plot; (**B**) loading plots. PC 1, principal component 1; PC 2, principal component 2.

Gas chromatography–mass spectrometry (GC‒MS) is a useful tool with high sensitive reproducibility and handling capacity for analysis via fast spectra accumulation times [19]. Through the GC‒TOFMS technique, the levels of primary metabolites including carbohydrates, amino acids, and organic acids, and secondary metabolites including phenolic compounds, were determined. A total of 47 metabolites were identified from four *L*. *platyphylla*). The metabolites identified in *L. platyphylla* were quantitated using selected ions and normalization, based on the signal intensity of IS (Tables S1 and S2). The quantitation data of the 47 hydrophilic metabolites were then subjected to principal component analysis (PCA), to explore the differences in metabolite profiles among the four *L. platyphylla* varieties. Metabolic profile analysis using the GC–MS technique and multivariate analysis have been used to explore metabolic differentiation among various genotypes. In particular, PCA has been successfully utilized as a preliminary stage in multivariate analysis to identify the patterns in complex experimental data [20]. The score plot from PCA results presented an overview of the differences among the four *L. platyphylla* cultivars, and the loading plot enabled correlation examination among the 47 metabolites (Figure 1). All the samples representing dissimilar colors were differently clustered, which were not differentiated by the two highest-ranking components. The components accounted for 64.1% of the total variance within the data set. Notably, the first component, accounting for 42.8% of the total variation, resolved the measured metabolite profiles of Cheongsim and Liriope Tuber No. 1 from the other two cultivars, Seongsoo and Cheongyanghaerae. This separation was mainly attributable to organic acids and amino acids; the corresponding loading vector showed that most of the organic acids, with the exception of three phenolic acids (*p*-hydroxybenzoic, vanillic, and sinapic acids), were predominant in Cheongism and Liriope cultivars, whereas the amino acids, with the exceptions of arginine and glutamine, differentiated the Seongsoo and Cheongyanghaerae cultivars from the other two. The PCA results showed that most amino acids were higher in Cheongyanghaerae compared with the other three cultivars, while Cheongsim had higher levels of organic acids than the other cultivars. 

**Figure 2:**
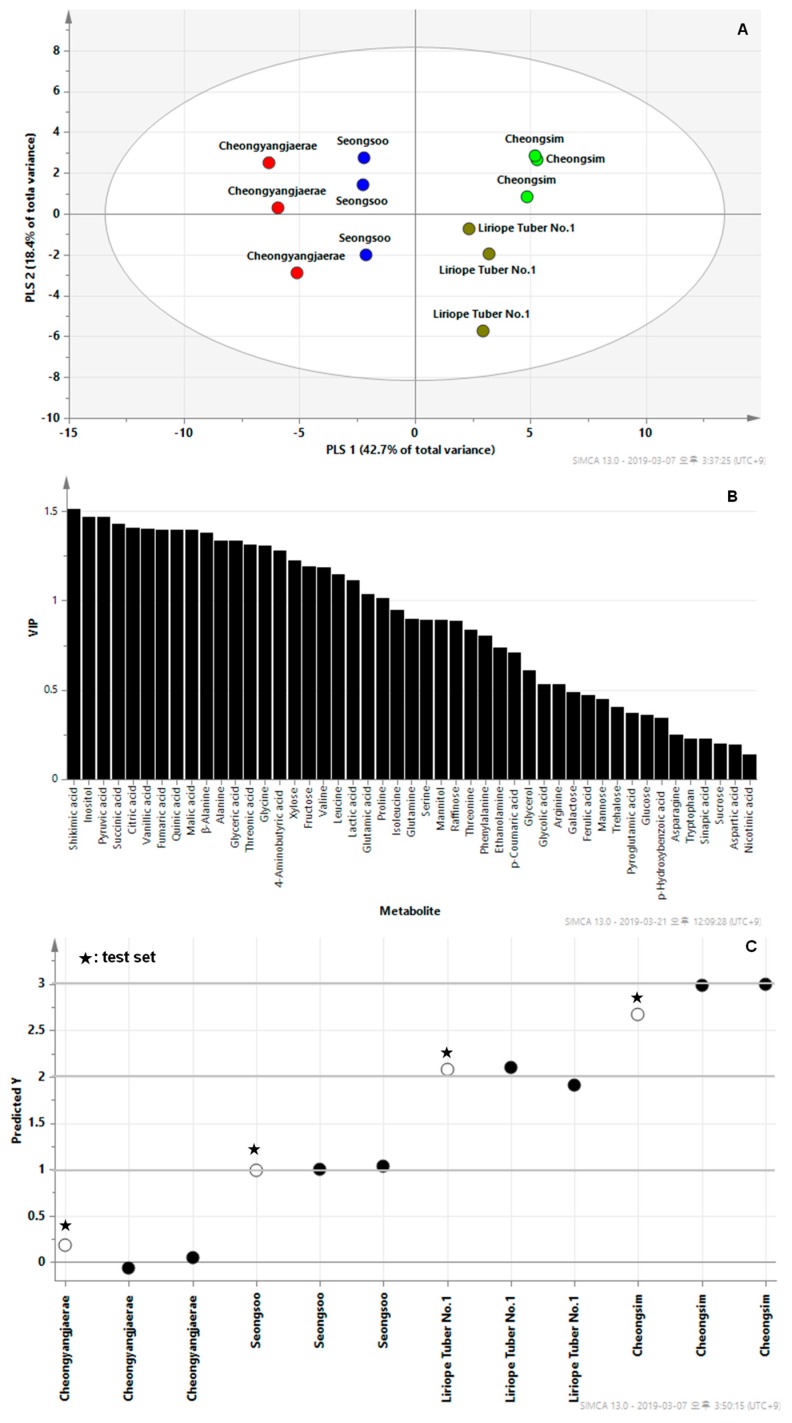
Score plots (**A**), variable importance in the projection (VIP) values (**B**), and external validation test (**C**) of partial least-squares discriminant analyses (PLS-DA) model derived from 47 metabolites from *L. platyphylla* samples.

To maximize the separation between the cultivars, we conducted partial least-squares discriminant analysis (PLS-DA) (Figure 2). PLS-DA is a well-established chemometric approach that separates groups of observations by rotating the PCA such that a maximum separation among classes, here *L. platyphylla* cultivars, is obtained. Identification of the first component aided in resolving the measured metabolite profiles of the four cultivars. The contribution of variables in the projection could be explained using variables important in the projection (VIP). Shikimic acid, one of the important building blocks employed in the biosynthesis of phenylpropanoids, was the most important for creating a prediction for *L. platyphylla* classification. The levels of shikimic acid and phenolic acids were higher in Cheongsim than other cultivars. For a cross validation, the total data should be divided into a training set and a test set. The 47 metabolites from the 12 samples were divided into 8 training set samples and 4 test set samples. The model shown in Figure 2C has Q_2_ = 0.949. In the partial least-squares (PLS) prediction model, a cross-validated correlation coefficient (Q_2_) > 0.9 indicates an excellent model.

**Figure 3:**
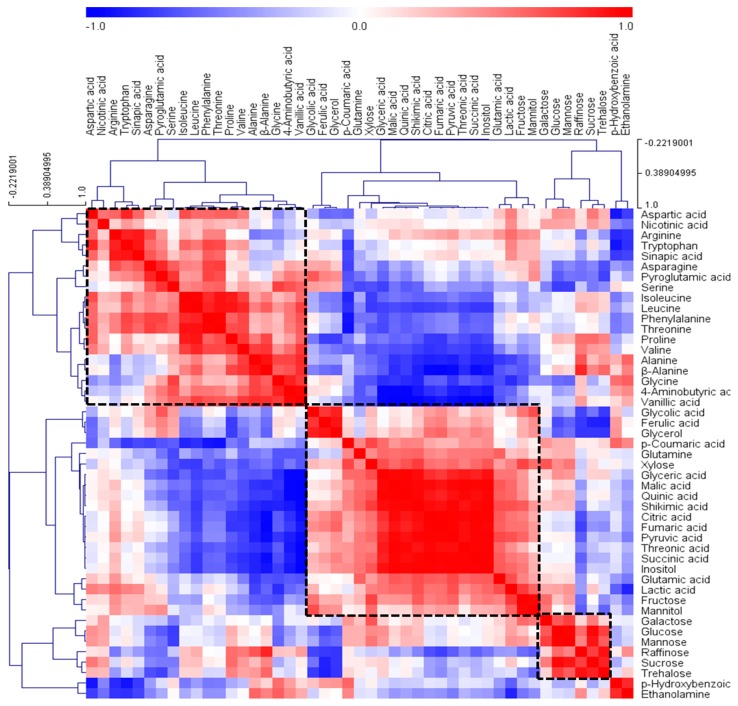
Correlation matrix and cluster analysis of results obtained from data on 47 metabolites for four cultivars of *L. platyphylla* Wang et Tang. Each square indicates the Pearson’s correlation coefficient for a pair of compounds, and the value for the correlation coefficient is represented by the intensity of the blue or red color as indicated on the color scale. Hierarchical clusters are represented by a cluster tree.

To gain insights into the relationships among the concentrations of the 47 hydrophilic metabolites identified in *L. platyphylla*, we performed a hierarchical cluster analysis (HCA) using Pearson’s correlation results on the data sets. Correlation analysis is one of the effective techniques to determine the strength of a relationship among quantitative samples. Using this method, significant correlations can be found between diverse metabolites involved in closely related pathways in a biological system [21]. The HCA results showed the degree of correlation among the identified metabolites. Compounds from the same class in a biological system containing amino acids, organic acids, or carbohydrates clustered together, which are marked by a dotted box in Figure 3. One group included all the amino acids except glutamine, whereas another group contained most organic acids, including the metabolites related to the TCA-cycle metabolism. The Pearson correlation coefficients among leucine, isoleucine, and valine, which are branched amino acids, were higher than 0.7. Similarly, a significant positive relationship was detected between serine and glycine (*r* = 0.7492, *p* = 0.005), and phenylalanine and tryptophan (*r* = 0.7284, *p* = 0.007), which are biosynthetically linked metabolites. Among the organic acids, the metabolites related to the TAC cycle, such as citric, succinic, fumaric, and malic acids, showed a strong correlation (*r* > 0.9, *p* < 0.0001). In addition, most of the identified carbohydrates, such as galactose, glucose, mannose, raffinose, sucrose, and trehalose, formed a group. However, fructose and xylose clustered with a group of organic acids rather than with other sugars. These results are in agreement with the loading plot of PCA results (Figure 1B), which described the robustness of the present experimental system and the potential of GC‒TOFMS‒based metabolic profiling, combined with chemometrics, as a useful tool for investigating metabolic links in biological systems.

Sugars, including xylose, fructose, mannose, galactose, glucose, sucrose, raffinose, and trehalose, were the most abundant metabolites in the four different cultivars. The total sugar level in Cheongsim was higher compared with the other cultivars; specifically, the levels of carbohydrates, including fructose, mannitol, sucrose, glucose, mannose, and galactose, were significantly higher in Cheongsim than in the other three cultivars. On the other hand, the levels of raffinose and trehalose were higher in Cheongyangjaerae. Liriope Tuber No. 1 showed the lowest level of total carbohydrates. Furthermore, a total of 18 amino acids were identified in all the cultivars, and the total amount of amino acids in Seongsoo was higher than in the other cultivars; however, the levels of only two amino acids, namely pyroglutamate and serine, were statistically higher compared with the other cultivars.

Similarly, the levels of proline, beta-alanine, and alanine were significantly higher in Cheongyangjaerae than in the other three cultivars. These results were consistent with a previous study, which suggested Cheongsim as a potential source of amino acids among the cultivars, based on HPLC-based amino acid analysis. A total of four intermediates of the tricarboxylic acid (TCA) cycle were detected in these varieties. Liriope Tuber No. 1 and Cheongsim contained larger pools of citrate, succinate, fumarate, and malate, which were related to the levels of pyruvate. Likewise, the levels of phenolic compounds, including vanillic acid, quinic acid, gallic acid, chlorogenic acid, caffeic acid, and benzoic acid, were higher in Liriope Tuber No. 1 and Cheongsim, compared with the other two cultivars.

## 3. Discussion

The vague boundary between primary and secondary metabolism, comprising the intermediate components biosynthesized by primary metabolism, which are also involved in secondary metabolism pathways, suggests interactions between both metabolisms. In this study, the connection between primary and secondary metabolites was investigated in four cultivars of *L*. *platyphylla* using GC–TOFMS and HPLC.

Carbohydrates play an important role in plant development, growth, and morphogenesis, and function as energy sources and metabolic precursors [22]. The lowest level of total carbohydrates in Liriope Tuber No. 1 reflected the requirement of carbons and energy to produce higher levels of spicatoside A. This finding is consistent with previous studies explaining the relationship between carbohydrates and secondary metabolites: Yeo et al. (2018) reported that the lower levels of carbohydrates in the sprouts of *Vigna unguiculata* exposed to blue light-emitting diodes reflected the higher production of phenolics and carotenoids [23]. Similarly, the total amount of carbohydrates in red flowers was lower than that in violet and white flowers of *Rhododendron schlippenbachii* due to their anthocyanin production [24]. Park et al.(2018) described that red lettuce contains lower levels of sugars and higher levels of phenolics and carotenoids than green lettuce [25], and that there is a negative correlation between carbohydrates and phenolic compounds in red radishes [6]. Furthermore, the enhancement of alkaloid production in cell cultures of the opium poppy, derived from treatment with a fungal elicitor, showed a more rapid depletion of carbohydrate pools [26]. Similarly, TCA intermediates play a major role in cellular catabolism and flavonol synthesis [27]. Therefore, the larger pools of TCA intermediates in Liriope Tuber No. 1 and Cheongsim suggest a greater accumulation of phenolic compounds. This finding is consistent with a previous study [20].

Amino acids are considered protein building blocks and are required for the biosynthesis of secondary metabolites, including phenolics and glucosinolates [28]. In this study, the Seongsoo cultivar contained the highest total amount of amino acids, which is consistent with a previous study reporting that the total amount of amino acids in Seongsoo was 1.76, 1.77, and 2.18 times higher than in Cheongyangjaerae, Cheongsim, and Liriope Tuber No. 1 cultivars, respectively [29].

Spicatoside A is a representative chemical found in *L*. *platyphylla*. In this study, spicatoside A was detected and quantified in four cultivars of *L*. *platyphylla*. Among them, Liriope Tuber No. 1 contained the highest amount of spicatoside A. Similarly, previous studies reported the identification of spicatoside A in *L*. *platyphylla* cultivated in South Korea; Cho et al. (1998) identified spicatoside A as well as spicatoside B using NMR [30]. Similarly, Shin (2002) detected and quantified both chemicals in Liriope Tuber No. 1 grown in South Korea using HPLC [31]. Furthermore, Lee et al. (2009) reported the changes in spicatoside A content in *L*. *platyphylla* tubers, with varying drying processes [32]. Choi et al. (2015) established the high performance counter-current chromatography (HPCCC) coupled with evaporative light scattering detection (ELSD) method for the separation of spicatoside A and spicatoside D from *L*. *platyphylla* [33].

In this study, the Liriope Tuber No. 1 cultivar had the highest level of spicatoside A, and the Cheongsim cultivar had the highest levels of phenolic compounds. On the other hand, the Seongsoo cultivar had the highest total amount of amino acids. Therefore, this study suggests that the Liriope Tuber No. 1 and Cheongsim cultivars could be a potential source of spicatoside A and phenolic compounds, respectively. As well, the Seongsoo cultivar could be considered a good source of amino acid for human intake. Furthermore, this study may help breeders establish a breeding strategy for new varieties with improved nutraceutical and nutritional qualities. 

## 4. Materials and Methods 

### 4.1. Plant Material

Four varieties of *L. platyphylla* (Seongsoo, Cheongsim, Cheongyangjaerae, and Liriope Tuber No. 1) were cultivated in a greenhouse at the Cheongyang Boxthorn Experiment Station, Cheongyang, South Korea. The tubers of the different varieties were harvested on 30 April 2016. The tubers were immediately placed in liquid nitrogen at −196 °C and then freeze-dried at −80 °C for at least 72 h (Ilshin Lab Co., Ltd., Daejeon, South Korea). Afterwards, mortars and pestles were used to grind the dried samples for further chemical analysis.

### 4.2. Spicatoside A Extraction and Quantification

Ten grams of dried sample powder was placed in a 250-mL flask and extracted with 80% MeOH (200 mL × 3) by reflux (80 °C, 30 min). After vortexing, the mixture was filtered using filter paper (Whatman No. 2) and left to stand for at least 1 h at room temperature. It was then centrifuged at 15,000 rpm at 4 °C for 5 min. Subsequently, the supernatant was evaporated in vacuo. The residue was dissolved in 1 mL of MeOH and filtered with a 0.45-μm pore-size hydrophilic polyvinylidene difluoride syringe filter (Ø 13 mm, Cat. no. 6779‒1304, Whatman Int. Ltd., Clifton, NJ, USA) into an HPLC vial. HPLC analysis of spicatoside A was performed on a Waters 1525 Binary HPLC (Miami, FL, USA) equipped with a photodiode array detector. Isolation was achieved on a SunFire C-18 stainless steel column (2.1 × 50 mm, 5 μm thickness) and absorption determinations were generated at 210 nm, with an oven temperature of 30 °C and a flow rate of 1.0 mL/min. A methanol (solvent A) and acetonaitrile (solvent B) isocratic solution (A:B (30:70), 15 min) was used. 

### 4.3. Phenylpropanoid Extraction and Quantification

Phenolic compounds were extracted using previously published procedures [34]. First, 0.2 g of dried sample powder was placed in a 15-mL tube and extracted with 80% MeOH (200 mL × 3). After sonication for 1 h, the mixture was filtered using filter paper (Whatman No. 2) and the extract was then evaporated. Next, the residue was dissolved in 2 mL of MeOH and filtered with a syringe filter into a brown vial. The HPLC analysis system, gradient program, and condition were carried out as detailed in a previous study [27]. We employed an NS-4000 HPLC system (Futecs, Daejeon, Korea), equipped with a C_18_ column (250 × 4.6 mm, 5 μm; RStech; Daejeon, Korea) and a UV−VIS detector. The conditions for HPLC–UV analysis were set as follows: injection volume, 20 μL; flow rate, 1.0 mL/min; detection wavelength, 280 nm; and oven temperature, 35 °C. The mobile phase consisted of a binary eluent of solvent A, acetic acid/water (0.2:99.8 v/v), and solvent B, methanol. Samples were eluted with the following gradient conditions: 0 min, 95% A; 4 min, 95–85% A; 9 min, 85% A; 14 min, 85–80% A; 24 min, 80% A; 54 min, 80–70% A; 55 min, 70–55% A; 65 min, 55% A; 75 min, 55–44% A; 77.0 min, 44–40% A; 79 min, 40% A; 80 min, 40–20% A; 90 min, 20% A; 91.0 min, 20–95% A; and 98.0 min, 95% A [34,35]. Quantitation was performed using the respective calibration curves. 

### 4.4. Metabolic Analysis

Extraction of hydrophilic compounds was carried out as described previously [36]. For the polar metabolite extraction, 10 mg of the powdered samples were measured and added to 1 mL of water:chloroform:methanol (2.5:1:1). Ribitol (60 µL, 0.2 mg/mL) was used as an internal standard (IS). Methoxime derivatization was executed through the addition of methoxyamine hydrochloride in pyridine (80 µL) and vigorous shaking for 90 min at 30 °C. Subsequently, trimethylsilyl etherification was carried out by adding *N*-methyl-*N*-trimethylsilyltrifluoroacetamide (80 μL). After incubation at 37 °C for 30 min, GC–TOFMS analysis was performed as described by Kim et al. (2013) [19]. The conditions for GC–TOFMS analysis were set as follows: split ratio, 1:25; injector temperature, 230 °C; flow rate of helium through the column, 1.0 mL/min; detector voltage, 1700 V; scanned mass range, 85–600 *m*/*z*; ion-source temperature, 200 °C; transfer line, 250. Furthermore, the temperature program was set as follows: initial temperature of 80 °C for 2 min, followed by an increase to 320 °C at 15 °C /min, and a 10 min hold at 320 °C. Leco ChromaTOF software was used for the peak detection and automated deconvolution of reference mass spectra. In-house libraries for standard compounds [35,36] and the National Institute of Standards and Technology (NIST) were used to identify the metabolite. The results were filtered with retention time, signal-to-noise ratio (>5:1), and mass spectral matching (based on a match >700). As a result, a total of 47 metabolites were identified (i.e., the metabolomics standards initiative (MSI) level 1) [37] (Table S2 and Figure S1). Concentration calculations of all metabolites were based on the ratios determined from the peak area of each metabolite/the peak area of the IS (Tables S1 and S3).

### 4.5. Statistical Analysis

Quantification data obtained from GC‒TOFMS were subjected to PCA and PLS-DA (SIMCA-P version 12.0; Umetrics, Umeå, Sweden) to assess the similarity between groups of multivariate data. The data file was scaled with unit-variance scaling (standardization) before all the variables were subjected to PCA. The PCA output is made up of score plots for visualizing the contrast between different samples and loading plots to describe the cluster separation. Furthermore, the SAS 9.4 software package (SAS Institute, Cary, NC, USA) was used for Pearson correlation analysis. Hierarchical clustering analysis and heat map visualization of the calculated correlation coefficients were executed using the software Multi-Experiment Viewer version 4.9.0 (http://www.tm4.org/mev/). The HPLC data were analyzed using SAS 9.4 software, applying an analysis of variance (ANOVA) evaluation and a Duncan’s multiple range test (DMRT).

## 5. Conclusions

To our knowledge, no previous studies have reported a comprehensive description of multiple metabolites in different cultivars of *L*. *platyphylla*. This study indicated that the Liriope Tuber No. 1 cultivar contained the highest level of spicatoside A and low amounts of carbohydrates, which are indicative of the carbon requirement and energy demand. Moreover, the observed high levels of TCA intermediates involved in flavonol metabolism were in agreement with the high levels of phenolic compounds. In this study, the Cheongsim cultivar contained the highest levels of phenolic compounds, while the highest total amount of amino acids was investigated in the Seongsoo cultivar. In contrast, the Liriope Tuber No. 1 cultivar contained the higher level of spicatoside A. Therefore, this study provides valuable information to help breed new cultivars with desired specific metabolite compositions.

## Figures and Tables

**Table 1:** Spicatoside A content in different cultivars of *Liriope platyphylla*.

No.	Cultivars	Contents (mg/g)
1	Liriope Tuber No. 1	1.83 ± 0.13 a ^1^
2	Cheongyangjaerae	1.25 ± 0.01 b
3	Seongsoo	1.01 ± 0.02 c
4	Cheongsim	1.09 ± 0.04 c

^1^ Different letters (a, b, c, d, respectively) indicate a significant difference at *p* < 0.05, applying a Duncan’s multiple range test.

**Table 2:** Phenolic compounds (µg/g dry weight) in different cultivars of *L. platyphylla.*

Contents (mg/g).	Liriope Tuber No. 1	Cheongyangjaerae	Seongsoo	Cheongsim
Gallic acid	29.07 ± 0.43 d ^1^	30.79 ± 1.37 c	33.54 ± 0.94 b	47.21 ± 0.30 a
Catechin	105.37 ± 0.43 a	105.65 ± 0.58 a	105.72 ± 0.12 a	105.54 ± 1.11 a
Chlorogenic acid	N.D	N.D	N.D	82.43 ± 1.39 a
Caffeic acid	38.17 ± 0.10 d	41.84 ± 0.54 c	42.89 ± 0.39 b	52.31 ± 0.76 a
Benzoic acid	220.44 ± 7.39 a	161.75 ± 5.12 d	191.78 ± 1.57 b	173.62 ± 2.51 c
Total	393.05 ± 7.87 b	340.03 ± 4.29 d	373.93 ± 0.74 c	461.11 ± 4.96 a

^1^ Different letters (a, b, c, d, respectively) indicate a significant difference at *p* < 0.05, applying a Duncan’s multiple range test.

## Supplementary Materials

The following are available online at https://www.mdpi.com/2218-1989/9/3/59/s1: Table S1: Metabolite peak height differences using GS–TOFMS; Table S2: Metabolites identified in GC–TOFMS chromatograms of *Liriope platyphylla*; Table S3: The raw data of metabolites obtained from *Liriope platyphylla* using GC–TOFMS. Figure S1: The chromatogram of metabolites obtained from *Liriope platyphylla* (Cheongyangjaerae) using GC–TOFMS. Peak identification: 1, pyruvic acid; 2, lactic acid; 3, alanine; 4, glycolic acid; 5, valine; 6, serine; 7, ethanolamine; 8, glycerol; 9, leucine; 10, isoleucine; 11, proline; 12, nicotinic acid; 13, glycine; 14, succinic acid; 15, glyceric acid; 16, fumaric acid; 17, threonine; 18, β-alanine; 19, malic acid; 20, aspartic acid; 21, pyroglutamic acid; 22, 4-aminobutyric acid; 23, threonic acid; 24, arginine; 25, glutamic acid; 26, phenylalanine; 27, p-hydroxybenzoic acid; 28, xylose; 29, asparagine; 30, vanillic acid; 31, glutamine; 32, shikimic acid; 33, citric acid; 34, quinic acid; 35, fructose; 36, galactose; 37, glucose; 38, mannose; 39, mannitol; 40, p-coumaric acid; 41, inositol; 42, ferulic acid; 43, tryptophan; 44, sinapic acid; 45, sucrose; 46, trehalose; 47, raffinose; IS, internal standard (ribitol).
