# Supplementary material for: Metabolic Analysis of Four Cultivars of Liriope platyphylla
Source: Metabolites. 2019 Mar 26;9(3):59. doi: 10.3390/metabo9030059 (PMC6468586; doi:10.3390/metabo9030059)
Supplement: Supplementary file 1 [file metabolites-09-00059-s001.zip › metabolites-455010-supp/190324_Metabolites_ Supplementary Figure S1.pdf]

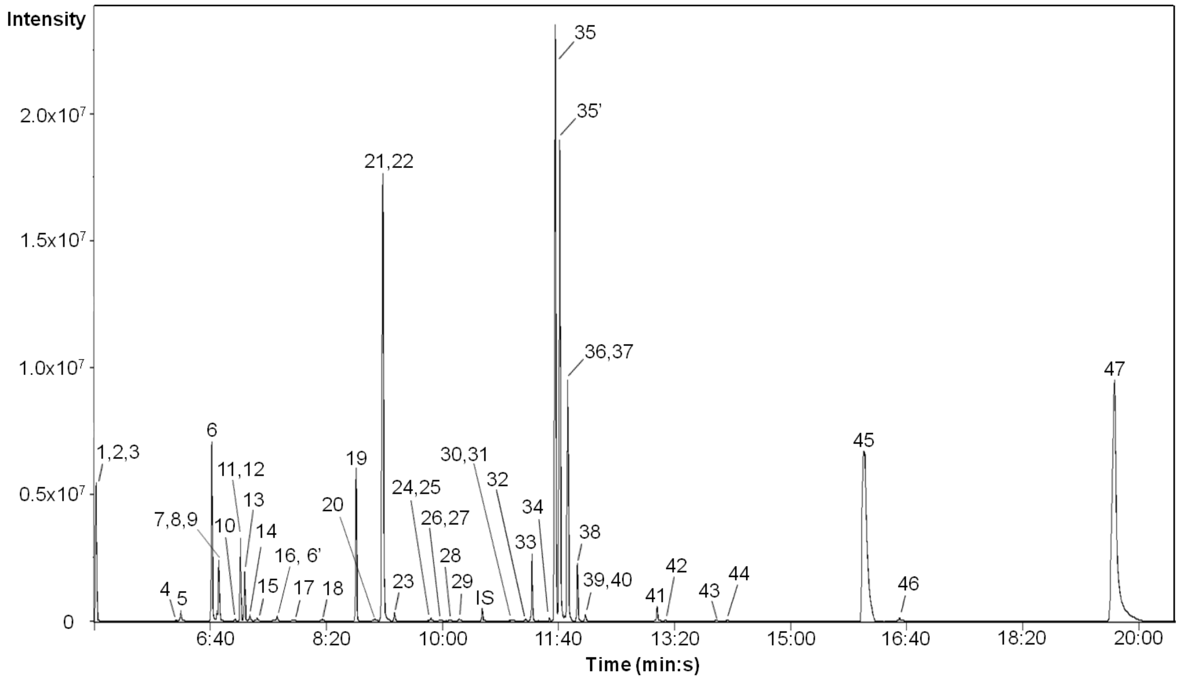

Figure S1. The chromatogram of metabolites obtained from *Liriope platyphylla* (Cheongyangjaerae) by using GC-TOFMS. Peak identification: 1, pyruvic acid; 2, lactic acid; 3, alanine; 4, glycolic acid; 5, valine; 6, serine; 7, ethanolamine; 8, glycerol; 9, leucine; 10. isoleucine; 11, proline; 12, nicotinic acid; 13, glycine; 14, succinic acid; 15, glyceric acid; 16, fumaric acid; 17, threonine; 18, β-alanine; 19, malic acid; 20, aspartic acid; 21, pyroglutamic acid; 22, 4-aminobutyric acid; 23, threonic acid; 24, arginine; 25, glutamic acid; 26, phenylalanine; 27, p-hydroxybenzoic acid; 28, xylose; 29, asparagine; 30, vanillic acid; 31, glutamine; 32, shikimic acid; 33, citric acid; 34, quinic acid; 35, fructose; 36, galactose; 37, glucose; 38, mannose; 39, mannitol; 40, p-coumaric acid; 41, inositol; 42, ferulic acid; 43, tryptophan; 44, sinapic acid; 45, sucrose; 46, trehalose; 47, raffinose; IS, internal standard (ribitol).
