# Supplementary material for: Metabolic Analysis of Four Cultivars of Liriope platyphylla
Source: Metabolites. 2019 Mar 26;9(3):59. doi: 10.3390/metabo9030059 (PMC6468586; doi:10.3390/metabo9030059)
Supplement: Supplementary file 1 [file metabolites-09-00059-s001.zip › metabolites-455010-supp/190324_Metabolites_ Supplementary Table S2.pdf]

Table S2. Metabolites identified in GC-TOFMS chromatograms of *Liriope platyphylla.*

¥	Compound	RT[1)]	RRT[2)]	Mass fragment[3)]	Quantification[4)]
1	Pyruvic acid	4.54	0.426	115, **174**, 189	174
2	Lactic acid	4.63	0.434	117, **147**, 191	147
3	Alanine	5.13	0.481	**116**, 147, 190	116
4	Glycolic acid	6.23	0.584	**147**, 177, 205	147
5	Valine	6.34	0.595	**144**, 156, 218	144
6	Serine	6.79	0.636	**116**, 132, 147	116
7	Ethanolamine	6.86	0.643	100, 147, **174**	174
8	Glycerol	6.89	0.646	103, 117, **147**	147
9	Leucine	6.90	0.647	102, 147, **158**	158
10	Isoleucine	7.12	0.667	147, **158**, 218	158
11	Proline	7.20	0.675	**142**, 158,216	142
12	Nicotinic acid	7.24	0.678	106, 136, **180**	180
13	Glycine	7.26	0.680	147, **174**, 248	174
14	Succinic acid	7.33	0.687	129, **147**, 247	147
15	Glyceric acid	7.43	0.697	133, **147**, 189	147
16	Fumaric acid	7.67	0.719	143, 147, **245**	245
17	Threonine	7.95	0.746	101, 117, **219**	219
18	β-alanine	8.37	0.785	147, **174**, 248	174
19	Malic acid	8.86	0.830	**147**, 233, 245	147
20	Aspartic acid	9.13	0.856	**100**, 147, 232	100
21	Pyroglutamic acid	9.24	0.866	147, **156**, 230	156
22	4-Aminobutyric acid	9.25	0.868	147, **174**, 304	174
23	Threonic acid	9.40	0.882	**147**, 205, 220	147
24	Arginine	9.90	0.928	**142**, 147, 162	142
25	Glutamic acid	9.93	0.931	128, 156, **246**	246
26	Phenylalanine	10.06	0.943	100, 192, **218**	218
27	p-Hydroxybenzoic acid	10.07	0.944	193, **223**, 267	223
28	Xylose	10.14	0.951	**103**, 147, 217	103
29	Asparagine	10.34	0.969	**116**, 132, 231	116
IS	Ribitol	10.67	1.000	103, 147, **217**	217
30	Vanillic acid	11.09	1.040	223, 267, **297**	297
31	Glutamine	11.11	1.042	147, **156**, 245	156
32	Shikimic acid	11.29	1.059	147, **204**, 255	204
33	Citric acid	11.39	1.068	147, **273**, 347	273
34	Quinic acid	11.63	1.090	**147**, 255, 345	345
35	Fructose	11.72	1.098	**103**, 147, 217	103
36	Galactose	11.85	1.111	**147**, 205, 319	147
37	Glucose	11.89	1.115	**147**, 160, 205	147
38	Mannose	12.03	1.128	**147**, 205, 319	147
39	Mannitol	12.15	1.139	**147**, 217, 319	319
40	p-Coumaric acid	12.33	1.156	**219**, 249, 293	219
41	Inositol	13.18	1.236	147, 217, **305**	305
42	Ferulic acid	13.30	1.247	308, 323, **338**	338
43	Tryptophan	14.03	1.315	**202**, 219, 348	202
44	Sinapic acid	14.19	1.331	**338**, 353, 368	338
45	Sucrose	16.12	1.512	147, **217**, 361	217
46	Trehalose	16.66	1.562	147, **191**, 361	191
47	Raffinose	19.78	1.854	204, **217**, 361	217

[1)] Retention time (min). [2)] Relative retention time (retention time of the analyte/retention time of the IS). [3)] Lists of first three ions with the highest intensity. Ions in bold indicate the most intense product ion [4)] Specific mass ion used for quantification.
